# SIRT7: a novel molecular target for personalized cancer treatment?

**DOI:** 10.1038/s41388-024-02976-8

**Published:** 2024-02-21

**Authors:** Alessandro Ianni, Poonam Kumari, Shahriar Tarighi, Thomas Braun, Alejandro Vaquero

**Affiliations:** 1 Chromatin Biology Laboratory, Josep Carreras Leukaemia Research Institute (IJC), Ctra de Can Ruti, Camí de les Escoles, Badalona, Barcelona, Catalonia 08916 Spain; 2 Department of Cardiac Development and Remodeling, Max-Planck-Institute for Heart and Lung Research, Bad Nauheim, 61231 Germany

**Keywords:** Oncogenes, Acetylation, Stress signalling

## Abstract

The Sirtuin family of NAD^+^-dependent enzymes assumes a pivotal role in orchestrating adaptive responses to environmental fluctuations and stress stimuli, operating at both genomic and metabolic levels. Within this family, SIRT7 emerges as a versatile player in tumorigenesis, displaying both pro-tumorigenic and tumor-suppressive functions in a context-dependent manner. While other sirtuins, such as SIRT1 and SIRT6, exhibit a similar dual role in cancer, SIRT7 stands out due to distinctive attributes that sharply distinguish it from other family members. Among these are a unique key role in regulation of nucleolar functions, a close functional relationship with RNA metabolism and processing -exceptional among sirtuins- and a complex multienzymatic nature, which provides a diverse range of molecular targets. This review offers a comprehensive overview of the current understanding of the role of SIRT7 in various malignancies, placing particular emphasis on the intricate molecular mechanisms employed by SIRT7 to either stimulate or counteract tumorigenesis. Additionally, it delves into the unique features of SIRT7, discussing their potential and specific implications in tumor initiation and progression, underscoring the promising avenue of targeting SIRT7 for the development of innovative anti-cancer therapies.

## Introduction

Dysregulation of sirtuins, a class of NAD^+^-dependent deacetylases, has emerged as a significant contributor to the development and progression of severe human diseases, encompassing conditions like diabetes, neurological disorders, cardiovascular diseases and cancer [1, 2]. The sirtuin family consists of seven members (SIRT1-SIRT7) in mammals, sharing a highly conserved catalytic domain while displaying substantial variations in their N-terminal and C-terminal regions [1, 2].

Sirtuins primarily serve as prominent deacetylases although some members also exert less characterized enzymatic functions such as a wider deacylase or mono-ADP ribosyltransferase activities [3–5]. These enzymes play pivotal roles in governing a wide range of biological functions encompassing cell proliferation, survival, genomic stability and regulation of metabolism. They accomplish this through epigenetic regulation of gene expression and chromatin dynamics either *via* direct deacetylation of specific histone marks or by regulating other histone modifiers. Furthermore, sirtuins exert control over a broad spectrum of non-chromatin targets, encompassing transcription factors and enzymes [6].

Sirtuins originally appeared in prokaryotes to participate in cobalamin biosynthesis [7] and later became crucial for cellular adaptation to varying of external conditions in different organisms. As external stimuli often modulate sirtuins catalytic activities, these molecules act as vital sensors of diverse stimuli and mediators of subsequent cellular responses [8]. For instance, due to their dependency on NAD^+^, Sirtuins can sense alterations in the metabolic state to activate signaling cascades involved in maintenance of cellular homeostasis [8].

**Fig. 1:**
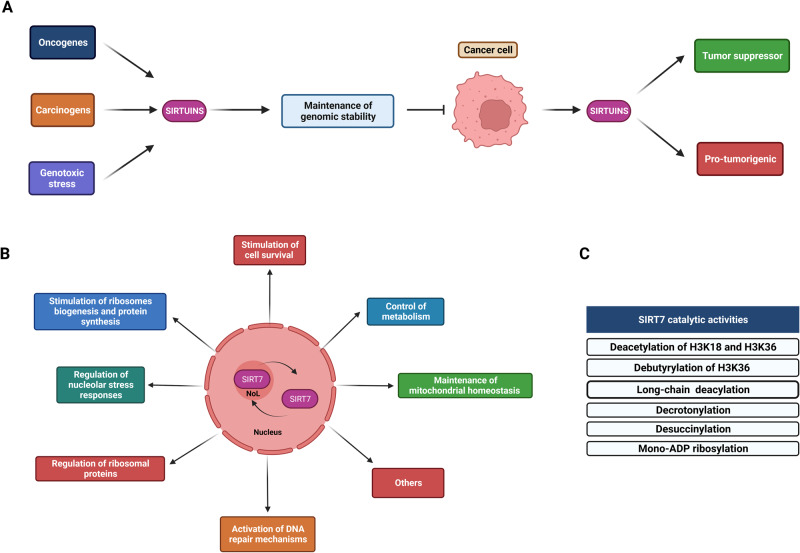
The complex role of Sirtuins in cancer and the unique molecular functions of SIRT7. **A** Sirtuins possess an intricate role in cancer initiation and progression. They counteract cell transformation mainly by ensuring maintenance of genomic stability following external stimuli. However, when cells acquired a malignant phenotype, Sirtuins appear to play both tumor suppressive and pro-oncogenic roles in a context-dependent manner. **B** SIRT7 is a nucleolar protein that shuttles between the nucleolus and the nucleoplasm and is involved in a plethora of fundamental biological functions as illustrated (NoL: nucleolus). **C** Scheme depicting the main enzymatic activities catalyzed by SIRT7.

The role of Sirtuins in cancer initiation and progression is notably intricate. They serve as pivotal agents ensuring the maintenance of genomic stability, particularly in response to genotoxic stress, mainly through activation of DNA repair mechanisms. In harmony with this, the depletion of certain Sirtuin members in mice results in accumulation of genomic instability and DNA damage, along with heightened susceptibility to carcinogen/oncogene-induced tumorigenesis [9–13] (Fig. 1A). The incidence of cancer rises in the elderly and remarkably, reduced expression/activity of some Sirtuins was observed in different tissues in both mice and humans during aging, suggesting that this event could potentially act as a precursor for development of cancer [14–16].

This collective evidence underscores the potent tumor suppressor role of these molecules. However, when cells have acquired a malignant phenotype, Sirtuins appear to exert both tumor suppressive or pro-oncogenic functions depending on the cellular context. Consistently, their expression levels associate with distinct clinical outcomes in specific malignancies [17, 18] (Fig. 1A).

## SIRT7 and the nucleolus: a unique functional interplay with considerable impact on tumorigenesis

SIRT7 distinguishes itself as the exclusive mammalian Sirtuin primarily localized within the nucleolus, a crucial organelle involved in ribosome biogenesis. In this compartment, SIRT7 cooperates with SIRT1 to uphold the stability of silenced rDNA genes in untransformed cells. This process involves maintaining proper heterochromatin structure at these genomic loci, thereby preventing homologous recombination of highly repeated rDNA sequences and avoiding global genomic instability. This mechanism represents a potential strategy adopted by SIRT7 to ensure genomic integrity and prevent cellular transformation and senescence [1, 19]. Interestingly, the maintenance of rDNA stability is a highly conserved function shared by SIRT1 and SIRT7, along with their homologous protein Sir2 in *Saccharomyces cerevisiae*, despite the extraordinary phylogenetic distance. In budding yeast, Sir2 is part of the RENT complex together with Net1 and Cdc14, and plays a crucial role in rDNA silencing and cell cycle control. In fact, loss of Sir2 is associated with rDNA instability, leading to shorten lifespan in this organism which is probably caused, at least in part, by accumulation of cytotoxic extrachromosomal rDNA circles (ERCs) that derive from recombination of the rDNA repeats [1, 20].

In contrast, in specific cancer cell types, SIRT7 appears to promote ribosome biogenesis by stimulating the transcription of active rDNA genes and the maturation of pre-ribosomal RNA (pre-rRNA) [1]. This function sharply differs from that of SIRT1, which inhibits this process [20]. Ribosome biogenesis assumes paramount importance to cater to the heightened biosynthetic and metabolic demands of rapidly proliferating cancer cells [21]. Hence, SIRT7 could potentially stimulate cell proliferation by enhancing ribosome biogenesis.

Nevertheless, SIRT7 assumes a pivotal role in repressing the expression of particular ribosomal proteins (RPs) [22, 23] and controls their incorporation into ribosomes [24]. Variations in specific RPs levels within the ribosomes can profoundly influence the translation of mRNA subsets involved in cancer progression [25]. Thus, in specific malignancies, SIRT7 may assume a complex role by stimulating global ribosomes biogenesis but altering the composition of ribosomes to influence the translation of specific mRNAs. SIRT7 also stimulates the expression of a specific subset of tRNAs [24], while it distributes to chromatin regions of genes involved in translation, potentially influencing their expression [22]. Thus, SIRT7 may influence translation of molecules involved in tumorigenesis by orchestrating a complex network of cellular reactions (Fig. 1B).

Compartmentalization of pro-oncogenic factors and tumor suppressors within the nucleolus emerged as a mechanism governing their activity or turnover in cancer cells [26]. Interestingly, SIRT7 interacts with several of these molecules including c-Myc, nucleophosmin (NPM1), Nucleolin and Mybbp [23, 27–29]. Thus, variations in SIRT7 expression across different malignancies could potentially play a pivotal role in some tumors by controlling nucleolar distribution of these molecules or specific interaction with other targets.

Additionally, the nucleolus has gained recognition as a crucial sensor of external stressors, mediating subsequent cellular stress responses. Various stress stimuli, including ultra violet (UV) irradiation, glucose deprivation and anti-cancer drugs treatments, facilitate the exclusion of nucleolar components, triggering the activation of downstream signaling cascades involved in cellular adaptation to the adverse conditions. These responses encompass activation of DNA repair mechanisms, inhibition of cell cycle progression and induction of apoptosis and are often mediated by activation of the tumor suppressor p53. This phenomenon is collectively termed the nucleolar stress response (NSR) [21]. Activation of the NSR in non-malignant cells especially following genotoxic stress can act as a safeguard mechanism to inhibit cellular transformation by preventing expansion of cells that have potentially acquired mutations. Conversely, anti-cancer drugs designed to trigger the NSR or/and inhibit ribosome biogenesis have been proposed as a novel strategy for inducing cancer cell death or halting proliferation [21]. SIRT7 plays a complex role in the NSR either by promoting the exclusion of nucleolar components from this compartment or by translocating itself. These reactions induce diverse effects including stimulation of DNA repair, inhibition of cell cycle progression or induction of cell survival, contingent upon the cellular context and the strength of the stimulus (Fig. 1B) [1, 30]. Notably, exclusion of SIRT7 from nucleoli reduces its capacity to stimulate ribosomes biogenesis [1], signifying the potential role of this event in curbing cell growth in response to specific treatments. Since, as we describe in detail below, SIRT7 often exert a pro-survival effect across different malignancies following anti-cancer treatments, investigating the precise influence of the nucleolar functions of SIRT7 on cancer progression and cellular responses to anti-cancer drugs presents an intriguing avenue for exploration of novel anti-cancer strategies.

## SIRT7 is a RNA-binding sirtuin with important roles in RNA synthesis and maturation

Another distinctive feature that sets SIRT7 apart from other sirtuins is its unique ability to engage in global RNA transcription, splicing, and RNA stability (Fig. 1B). In sharp contrast to other sirtuins, SIRT7 possesses the capacity to bind to RNA. This distinctive trait, as elaborated below, plays a fundamental role in stimulating its catalytic activity [31]. Binding of SIRT7 to RNA may influence RNA functions. For instance, SIRT7 deacetylates N4-acetylcytidine to control RNA stability and translation efficiency [32]. Fascinatingly, while the majority of RNA associated with SIRT7 comprises rRNA, a noteworthy fraction includes mRNA and non-coding RNA involved in protein translation and chromatin regulation [31]. This suggests that SIRT7 could significantly influence signaling cascades inherently linked to cancer progression by governing the stability of specific subsets of RNA. Furthermore, given that specific stress conditions prevalent in cancer cells reduce SIRT7 accumulation in the nucleolus, it is conceivable that, under such circumstances, SIRT7 may specifically interact and control distinct RNAs to control tumor progression. Further studies are required to substantiate this assertion.

SIRT7 also participates in mRNA splicing. Indeed, it controls the activity of the alternative splicing factor PHF5A, an event that leads to decreased gene expression due to retained intron-induced abnormal alternative splicing [33]. Moreover, SIRT7 interacts with molecules involved in mRNA processing, further supporting a critical role in RNA maturation [34]. SIRT7 also exerts a profound impact on RNA polymerase II (Pol II)-dependent transcription through multiple mechanisms. Firstly, it deacetylates CDK9, a subunit of the elongation factor P-TEFb. This event enhances CDK9-mediated phosphorylation of the C-terminal domain (CTD) of Pol II, to promote transcriptional elongation [34]. Secondly, SIRT7 prevents R-loops-mediated stalling of RNA polymerase by deacetylating the DEAD (Asp-Glu-Ala-Asp)-box RNA helicase DDX21. This event boosts DDX21 activity, facilitating the resolution of R-loops and thereby promoting transcriptional elongation. Additionally, since aberrant persistence of R-loops promotes accumulation of DNA damage, SIRT7 ensures the maintenance of genomic stability through this mechanism [35]. Given the established association of aberrant RNA transcription and maturation with tumorigenesis [36], further investigations will unveil the extent of SIRT7’s influence on these processes in the context of cancer.

## SIRT7 possesses unique catalytic activities with potential impacts in cancer progression

While SIRT7 shares common catalytic activities with other sirtuins, such as removal of acetyl and other acyl groups, it also demonstrates the ability to catalyze distinct reactions on specific targets.

SIRT7 demonstrates a remarkable specificity for deacetylating particular histone marks to ensure epigenetic regulation of gene expression. One of its notable actions involves the deacetylation of lysine 18 on histone 3 (H3K18), a modification also targeted by SIRT1 and SIRT6 [37]. However, specific distribution of SIRT7 to the chromatin may assume a unique role in the epigenetic control of expression of a distinct subset of genes. SIRT7-mediated deacetylation of H3K18 activates pro-oncogenic processes across different types of cancers by epigenetically silencing the expression of key tumor suppressor genes as described below [22, 38–40].

SIRT7 stands out for its remarkable deacetylase and, to a lesser extent, debutyrylation activity toward H3K36, which is negligible for other sirtuins (Fig. 1C) [37, 41]. The contribution of acetylation and butyrylation of these residues in chromatin regulation and gene expression expression in cancer is largely unknown. In contrast, methylation of H3K36 (H3K36me) appears to control indispensable chromatin functions encompassing epigenetic regulation of gene expression, and splicing. Intriguingly, diminished H3K36me, whether due to mutations in this residue or neighboring regions, or resulting from impaired activity of specific writers (methyltransferases), has been linked to heightened tumorigenesis [42]. As methylation, acetylation/butyrylation at a specific lysine residues are mutually exclusive events, SIRT7 may inherently control H3K36me through its activity ultimately influencing cancer progression at least in specific malignancies. Further research is warranted to support this claim.

SIRT7 also catalyzes the removal of other acyl groups such as succinylation and crotonylation [33, 43, 44], two activities that have been attributed mainly to mitochondrial Sirtuins [43, 45, 46]. Thus, due to distinct subcellular localization, SIRT7 may control nuclear and nucleolar targets through this activity. SIRT7 desuccinylates both histones and non-histone targets [47, 48]. SIRT7-mediated desuccynylation of H3K122 plays a fundamental role in promoting DNA repair under stress [47]. However, as H3K122 succynylation is paramount in regulating transcription, SIRT7 may act as a central player in controlling gene expression in cancer through this reaction [44]. Additionally, as detailed below, SIRT7-mediated desuccynilation of the non-histone target PRMT5 appears indispensable for driving progression of liver cancer [48]. In contrast, the impact of SIRT7-dependent decrotonylation activity in cancer remains unexplored (Fig. 1C).

SIRT7 also exhibits the capacity to catalyze the hydrolysis of long-chain fatty acyl lysine, and intriguingly, this activity is notably enhanced when SIRT7 binds to RNA, particularly ribosomal and transfer RNA [31]. Lysine long-chain fatty acylation frequently facilitates the membrane localization of substrate proteins, thereby governing the secretion and activation of specific cell signaling cascades that can influence cancer progression [49]. While the complete characterization of the molecular targets of SIRT7 for this activity is yet to be accomplished, the potential impact of SIRT7 on cancer progression through this reaction is evident. Moreover, in cancer cells, where numerous oncogenes stimulate rRNA transcription [21], the increased availability of rRNA may aberrantly activate SIRT7 functions. This event may synergize with altered SIRT7 expression that often occurs in cancer cells as described below, ultimately contributing to cancer progression.

Another intriguing aspect of SIRT7 is its role as a prominent mono-ADP-ribosyltransferase, exhibiting the capability for autoregulation through this enzymatic activity [5]. Auto-mono-ADP ribosylation of SIRT7 controls chromatin distribution of SIRT7 especially in response to glucose starvation by facilitating SIRT7 interaction with the macro domain of the histone variant macroH2A1. This interaction also regulates macroH2A1 enrichment to intragenic regions and gene promoters to ultimately control gene expression [5]. Thus, conditions of nutrient scarcity, often occurring in cancer cells, may activate the SIRT7-macroH2A1 axis in cancer cells potentially influencing cancer progression. Given that dysregulated expression of macroH2A histone variants often occurs in tumors [50], alterations of this pathway in specific malignancies should play relevant roles in tumor initiation and progression. Moreover, SIRT7 could mono-ADP-ribosylate other still unknown targets to influence their functions in cancer (Fig. 1C).

## The complex contribution of SIRT7 to distinct malignancies

In this section, we provide a comprehensive overview of the molecular mechanisms, discovered thus far, employed by SIRT7 to control initiation and progression of different malignancies.

### Role of SIRT7 in cancers of the digestive system

Digestive system tumors encompass a diverse range of malignancies affecting organs within the digestive tract, such as the liver, intestine, pancreas, and esophagus. These tumors collectively contribute to approximately 26% of the global cancer incidence and are responsible for nearly 35% of all cancer-related deaths [51, 52].

SIRT7 emerges as a significant driving force behind the progression of these malignancies. Consistently, SIRT7 levels exhibit an observable increase in liver [53], pancreas [38], gastric cancer [54, 55] and colorectal tumors [56] as compared to healthy counterparts. High levels of SIRT7 in these malignancies associate with a more aggressive phenotype and correlate with the stage of tumor progression. Moreover, inhibition of SIRT7 in cells derived from these tumors induces a significant deceleration in cancer cell growth, accompanied by a hindered capacity for tumor formation in mouse xenograft models [38, 56–59].

A complex interplay of mechanisms underpins the heightened expression of SIRT7 in some of these tumors. This includes downregulation of prominent tumor suppressor microRNAs targeting SIRT7, upregulation of circular RNAs sponging these molecules [53, 57, 60] or deregulation of specific factors controlling SIRT7 protein stability [38].

**Fig. 2:**
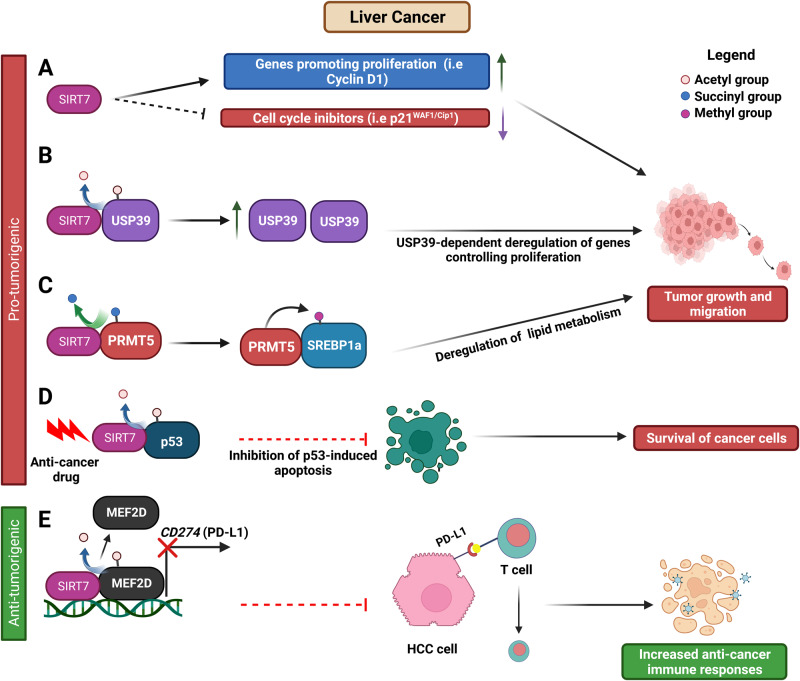
Molecular mechanisms employed by SIRT7 to promote or counteract liver cancer progression. See text for details.

SIRT7 employs different mechanisms to facilitate progression of these tumors. In hepatocellular carcinoma (HCC), the most prevalent form of liver cancer [61], SIRT7 spurs the advancement of the G_1_/S cell cycle phase by stimulating the expression of favorable drivers of cell cycle progression such as Cyclin D1 while concurrently repressing cell cycle inhibitors such as p21^WAF1/Cip1^ (Fig. 2A and Table 1) [57]. Furthermore, SIRT7 increases the stability of USP39 by means of direct deacetylation. USP39 is a pivotal factor involved mRNA splicing with recognized oncogenic functions in HCC. SIRT7-mediated stabilization of USP39 enables SIRT7 to orchestrate the expression of genes implicated in tumorigenesis, thus effectively fostering cancer progression (Fig. 2B and Table 1) [58]. SIRT7 also engages in the desuccinylation of arginine methyltransferase 5 (PRMT5), thereby promoting PRMT5-mediated methylation of the transcription factor SREBP1a. This cascade of events culminates in the disruption of SREBP1a’s governance over genes implicated in lipid biosynthesis, fueling the acceleration of cancer cell proliferation, migration, and metastasis formation (Fig. 2C and Table 1) [48]. SIRT7 assumes a pivotal role in safeguarding the survival of HCC cells in response to anti-cancer drugs predominantly through direct deacetylation of the tumor suppressor p53, thereby hampering p53-driven apoptotic pathway (Fig. 2D and Table 1) [62]. Remarkably, the impact of SIRT7 on p53 activation seems to be contingent only on specific cellular contexts and stimuli. Indeed as described above, in specific cellular contexts, SIRT7 appears to rather activate the p53 response by activating the NSR, while in others SIRT7 lacks its capacity to bind and deacetylate p53 [30]. As p53 is frequently deleted or mutated across various malignancies, SIRT7’s role in cancer likely hinges on its ability to modulate p53 and the genetic status of the p53 gene.

**Table 1:**
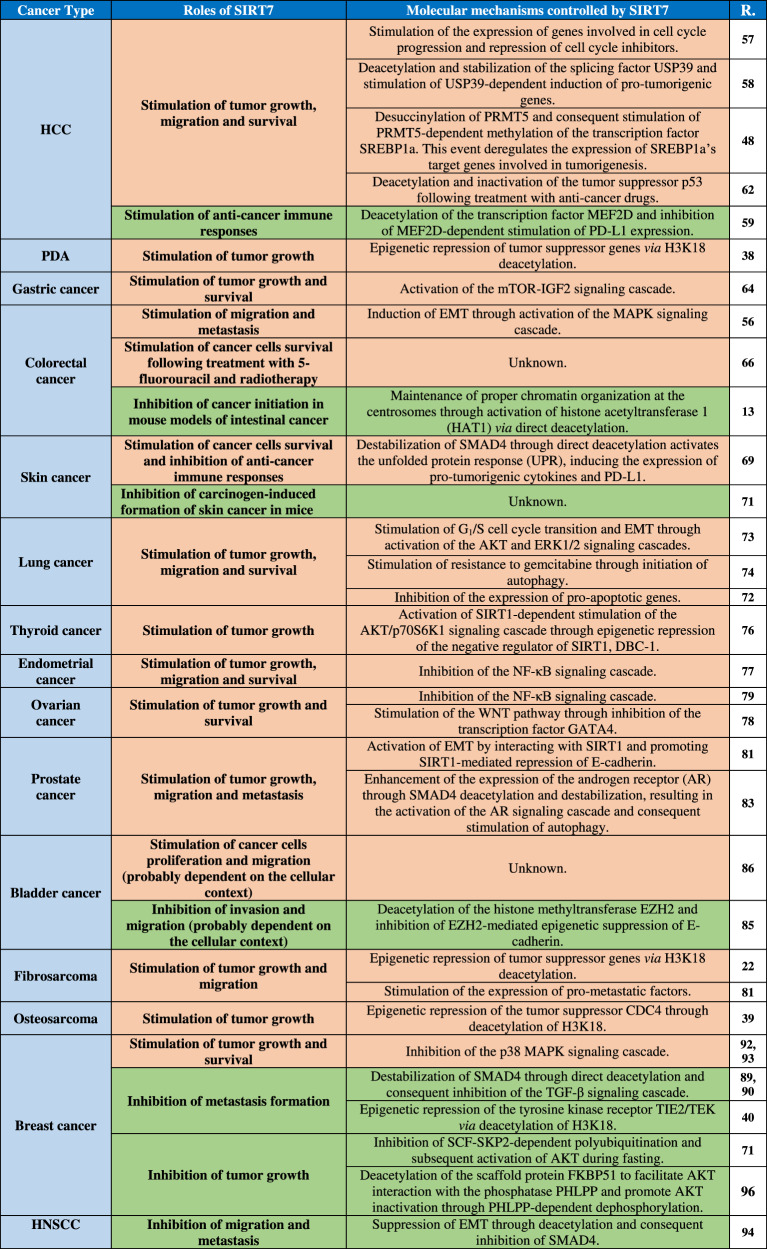
Main roles of SIRT7 in controlling cancer initiation and progression.

Red highlights denote pro-tumorigenic effects, while green highlights represent anti-tumorigenic functions. References are denoted by R.

*HCC* hepatocellular carcinoma, *PDA* pancreatic ductal adenocarcinoma, *HNSCC* head and neck squamous cell carcinoma, *EMT* epithelial-to-mesenchymal transition.

In line with its pro-oncogenic functions in HCC, novel specific inhibitors of SIRT7 demonstrated capacity to retard growth of liver cancer cells in in vivo, suggesting that targeting SIRT7 may represent an efficient therapeutic target for this devastating malignancy [63].

Nevertheless, beyond its role in fostering the growth and survival of HCC cells, SIRT7 emerges as a pivotal participant in the elicitation of anti-cancer immune responses, which can potentially impede cancer progression. In fact, SIRT7 reduces the levels of acetylation of the transcription factor myocyte enhancer factor 2D (MEF2D), thereby impairing recruitment of MEF2D at the promoter of the *CD274* gene. This event leads to a decline in *CD274* gene expression within HCC cells. *CD274* encodes for Programmed cell death 1 ligand 1 (PD-L1), a molecule whose interaction with a receptor expressed on immune system cells triggers a signaling cascade that dampens the immune response. Thus, through this mechanism, SIRT7 prevents cancer cells from evading immune surveillance (Fig. 2E and Table 1). Based on this evidence, it was proposed that combined application of SIRT7 inhibitors together with blockage of the PD-L1 pathway may represent a strong therapeutic intervention for treatment of HCC [59].

**Fig. 3:**
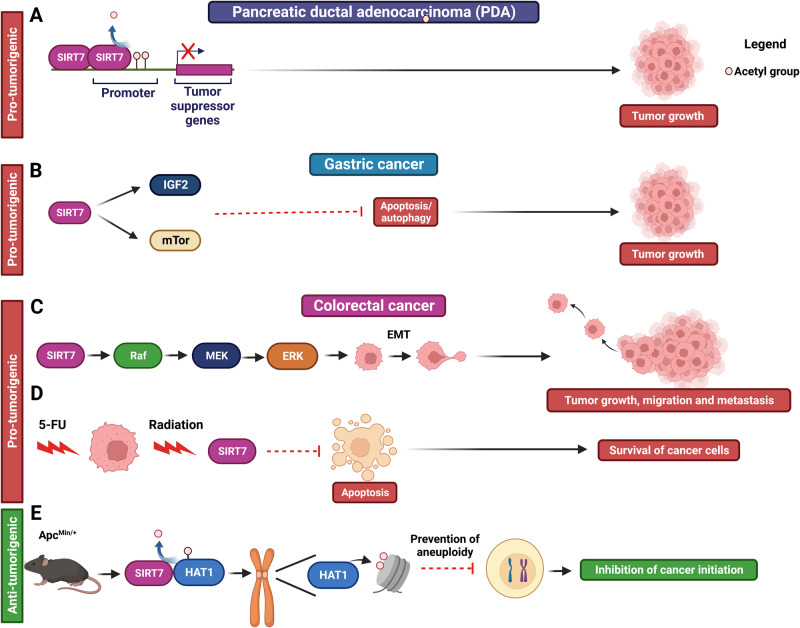
Role of SIRT7 in cancers of the digestive system. Molecular mechanisms employed by SIRT7 to control initiation and progression of pancreatic (**A**), gastric (**B**) and colorectal cancer (**C–E**).

SIRT7 appears to promote proliferation and survival of pancreatic ductal adenocarcinoma (PDA) by epigenetically repressing expression of tumor suppressors through H3K18 deacetylation (Fig. 3A and Table 1) [38].

In gastric cancers, SIRT7 inhibits apoptosis and autophagy to ensure survival of cancer cells by activating the mTOR-IGF2 signaling cascade (Fig. 3B and Table 1) [64]. Given the clear role of mTOR activation in the stimulation of ribosome biogenesis and protein synthesis [65], it is intriguingly to speculate that in this type of malignancy SIRT7 may sustain cancer cells growth by stimulating these functions.

SIRT7 emerges as a pro-tumorigenic factor in colorectal cancer as well. Within these tumor contexts, SIRT7 heightens the activation of the MAPK signaling cascade, thereby triggering epithelial-to-mesenchymal transition (EMT) and ultimately fostering migratory potential, promoting the formation of metastasis (Fig. 3C and Table 1) [56]. Furthermore, SIRT7 assumes a vital function in bolstering the viability of cancer cells upon exposure to the combined treatment of 5-fluorouracil (5-FU) and radiotherapy – a prevailing therapeutic modality targeting this malignancy. These insights underscore the possibility of utilizing SIRT7 inhibition to heighten the responsiveness of cancer cells to chemoradiation (Fig. 3D and Table 1) [66]. Interestingly, in the context of colorectal cancer treatment, the administration of 5-FU has been observed to trigger the production of fluorinated ribosomes [67]. These altered ribosomes enhance the translation of specific mRNA molecules, promoting the survival of cancer cells [67]. Given SIRT7’s involvement in regulating ribosomal composition, it becomes intriguing to investigate whether altered SIRT7 expression, when combined with 5-FU treatment, could potentially lead to distinct outcomes in mRNA translation. These differential translation patterns might contribute to varying effects of chemotherapy in the context of this particular malignancy.

Nevertheless, notwithstanding the evident pro-tumorigenic influence of SIRT7, a recent study presents an intriguing counterpoint. Ablation of *SIRT7* in the murine model of intestinal cancer adenomatous polypolis coli (Apc^Min/+),^ remarkably accelerates tumorigenesis [13]. Within the context of the intestinal epithelium, SIRT7 plays a key role in upholding genomic stability by stimulating the enzymatic activity of histone acetyltransferase 1 (HAT1) *via* direct deacetylation. Consequently, SIRT7 deficiency results in diminished HAT1-mediated histone acetylation, culminating in the destabilization of chromatin at centrosomes and consequent aneuploidy that propels cancer initiation (Fig. 3E and Table 1) [13].

These data clearly highlight the intricate duality of SIRT7 in cancer initiation and progression.

### SIRT7 in skin cancer

Skin cancer is broadly categorized into melanoma and non-melanoma tumors, contingent upon the cell of origin (melanocytes or epidermal cells, respectively) [68].

**Fig. 4:**
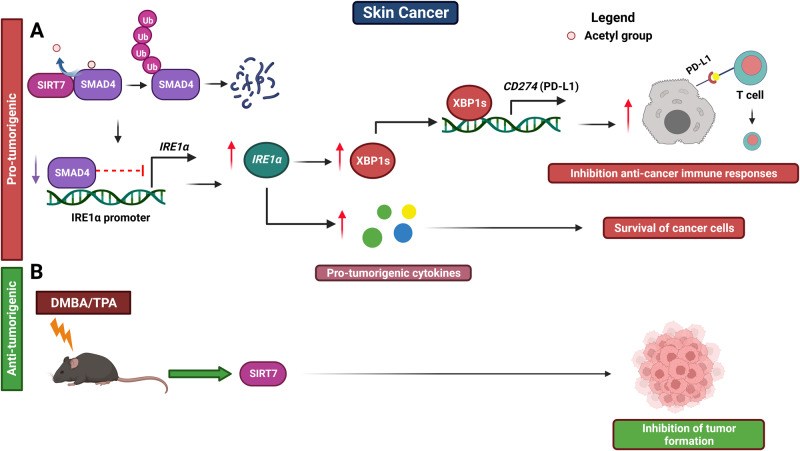
Role of SIRT7 in skin cancer initiation and progression. See text for details.

SIRT7 assumes a pivotal pro-tumorigenic role in melanoma by enhancing various aspects of tumor development including growth, survival, migration and evasion from anti-cancer immune responses [69, 70]. This is accomplished, at least in part, by activating the unfolded protein response (UPR) [69]. In the harsh tumor microenvironment, circumstances of nutrient and oxygen scarcity arise, culminating in hypoxia, acidosis, and endoplasmic reticulum (ER) stress. Consequently, the UPR is activated to restore ER functions and support the survival of cancer cell [69]. When ER stress occurs, the endoribonuclease activity of IRE1α, a trans-membrane receptor of the ER, prompts the splicing of X-box binding protein 1 (XBP1) mRNA. This mRNA encodes a molecule that initiates a transcriptional program that stimulates the expression of factors responsible for protein folding and pro-tumorigenic cytokines. Activation of this pathway also culminates in a dampened anti-cancer immune response by increasing the binding of XBP1s to the *CD274* promoter, encoding for PD-L1. This sequence of events ultimately triggers XBP1s-mediated PD-L1 expression, promoting evasion of melanoma cells from anti-cancer immune responses. SIRT7 appears to stimulate the IRE1α-XBP1 pathway in melanoma cells by deacetylating and destabilizing SMAD4. This event reduces SMAD4 engagement with the IRE1α promoter, thereby alleviating SMAD4’s repressive impact on IRE1α expression (Fig. 4A and Table 1). Aligning with these discoveries, inhibition of SIRT7 has demonstrated the capability to amplify the impact of cancer immunotherapy that incorporates anti-PD-L1 antibodies [69]. It is worth to noticing, that the effect of SIRT7 in controlling PD-L1-mediated immune responses strikingly diverges from HCC, where SIRT7 actually suppresses PD-L1 expression by inhibiting the MEF2D transcription factor [59]. However, in melanoma cells, SIRT7 seems to function independently of MEF2D [69], underscoring its ability to exert opposing effects on the same molecular pathways depending on the specific context.

However, SIRT7 seems to manifest a safeguarding function against skin cancer initiation in mice. This is evidenced by the fact that *SIRT7* heterozygous mice, subjected to the two-stage DMBA/TPA-induced skin cancer model, display a greater development of skin papillomas compared to their wild-type counterparts (Fig. 4B and Table 1) [71]. Furthermore, SIRT7 appears to activate the tumor suppressor p53 in the skin following exposure to UV irradiation, a main driver of skin cancer, by triggering the NSR. This evidence suggests that SIRT7 may exert tumor-suppressive roles in cancer initiation by controlling this mechanism although further research is warranted to support this claim [29].

### SIRT7 in lung cancer

**Fig. 5:**
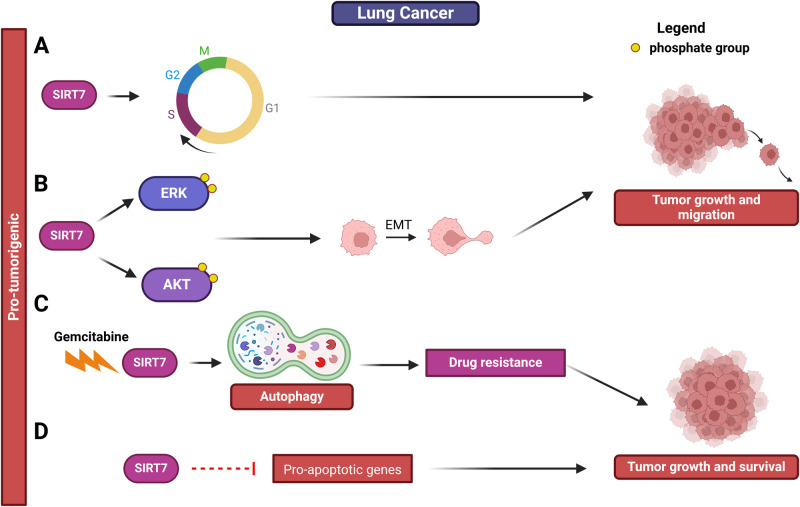
Role of SIRT7 in lung cancer progression. See text for details.

SIRT7 exerts pro-tumorigenic functions also in lung cancer and enhanced SIRT7 levels were found in this malignancy compared with healthy tissues counterparts [72, 73]. SIRT7 promotes proliferation and migration of lung cancer cells both in vitro and in vivo by stimulating G_1_/S cell cycle transition and promoting EMT mainly through activation of the AKT and ERK1/2 signaling cascades (Figs. 5A, B and Table 1) [73]. SIRT7 also bolsters the survival of lung cancer cells in response to chemotherapy. Consistently, inhibition of SIRT7 renders lung cancer cells highly susceptible to the effects of gemcitabine, a widely employed antimetabolite in lung cancer treatment. Mechanistically, SIRT7 prompts the initiation of autophagy as a response to gemcitabine, a process known to drive resistance to this drug (Fig. 5C and Table 1) [74]. Since the activation of the NSR was recently linked to autophagy [75], it will be fascinating to investigate whether this effect is, to some extent, reliant on regulation of the NSR. Furthermore, SIRT7 represses the expression of pro-apoptotic genes, promoting the survival of lung cancer cells and facilitating tumor growth (Fig. 5D and Table 1) [72]. Thus, targeting SIRT7 in lung cancer cells offers a prospective strategy to overcome drug resistant cancers [74].

### SIRT7 in thyroid cancer

**Fig. 6:**
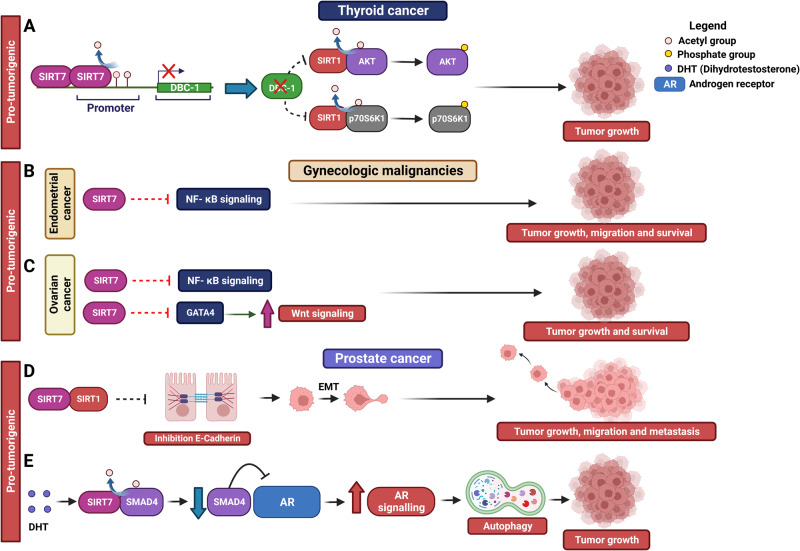
Role of SIRT7 in indicated malignancies. Molecular mechanisms employed by SIRT7 to control progression of thyroid cancer (**A**), gynecologic malignancies (**B**, **C**) and prostate cancer (**D**, **E**).

The contribution of SIRT7 to thyroid cancer progression is closely linked to its interaction with another sirtuin family member: SIRT1. Elevated SIRT7 levels within these tumors intensify its epigenetic repression of Deleted in breast cancer 1 (DBC-1), a robust inhibitor of SIRT1. Consequently, SIRT7 propels the activation of SIRT1, initiating SIRT1-mediated deacetylation and activation of the AKT/p70S6K1 signaling cascade that in turn stimulates the growth and survival of cancer cells (Fig. 6A and Table 1) [76].

### SIRT7 in gynecologic malignancies

SIRT7 appears to act as a pro-tumorigenic factor also in gynecologic malignancies. Elevated expression of SIRT7 has been detected in endometrial and ovarian cancer when compared with healthy tissues [77, 78]. Moreover, inhibition of SIRT7 in endometrial and ovarian cancer cells leads to diminished cell proliferation, migration and higher sensitivity to anti-cancer drugs treatments [77, 79]. This beneficial outcome is associated with elevated activation of the nuclear factor-κB (NF-κB) signaling cascade, suggesting that SIRT7 inhibits this pathway (Figs. 6B, C and Table 1) [77, 79]. Interestingly, the sequestration of key molecules involved in the activation of the NF-κB cascade in the nucleolus has been recognized as a critical event controlling this pathway [80]. Further research will reveal whether regulation of this signaling cascade by SIRT7 involves its contribution into nucleolar functions.

Finally, in ovarian cancer, SIRT7 restrains the activity of the transcription factor GATA4 to effectively activate the Wnt signaling cascade and stimulate cancer cells growth **(**Fig. 6C and Table 1) [78].

### SIRT7 in prostate cancer

In a manner reminiscent of its function in other cancer types, SIRT7 has risen to prominence as a key influencer in propelling the advancement of prostate cancer (PCa). Evidently, SIRT7 levels exhibit an elevation within PCa in contrast to healthy tissues. Furthermore, heightened SIRT7 expression aligns with escalated aggressiveness, increased metastatic potential, and an overall less promising prognosis [81–83]. Ablation of SIRT7 in PCa cells diminishes tumor growth, curbs cell migration, and attenuates the formation of metastases in mouse xenograft models [81, 83]. Furthermore, SIRT7 seems to incite drug resistance and dampen radiation sensitivity within these tumors [82, 83]. At the mechanistic level, SIRT7 interacts with SIRT1, promoting SIRT1-induced repression of E-cadherin. This sequence of events propels the initiation of EMT, although the precise operational details remain unexplored (Fig. 6D and Table 1) [81]. In addition, when faced with androgen stimulation, SIRT7 prompts the initiation of autophagy by enhancing the expression of the androgen receptor (AR), consequently initiating the downstream signaling cascade. This mechanism involves SIRT7-mediated deacetylation and subsequent destabilization of SMAD4, a key negative regulator of AR expression, resulting in an augmented expression of AR [83] (Fig. 6E and Table 1).

### SIRT7 in bladder cancer

**Fig. 7:**
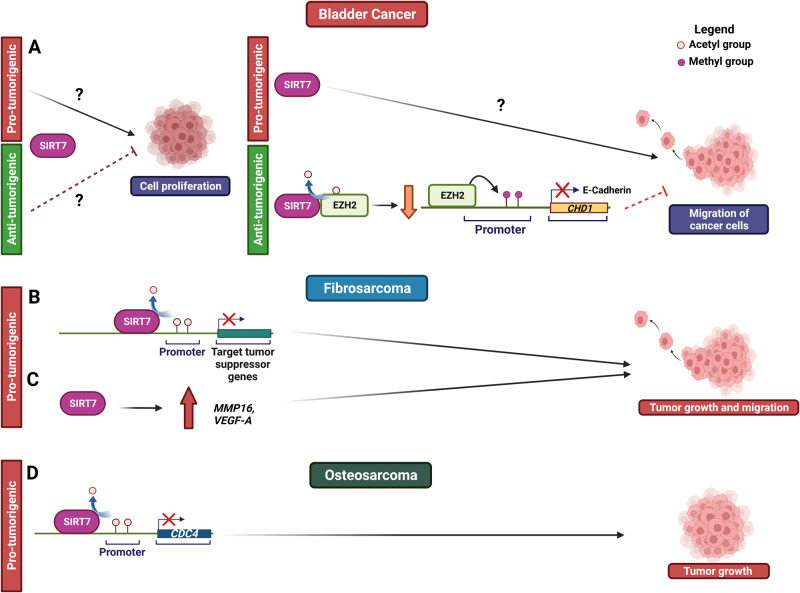
Molecular mechanisms underpinning SIRT7’s role in bladder cancer and sarcoma progression. See text for details.

Bladder cancer (BCa) stands as the prevalent malignancy within the urinary tract, carrying a dismal prognosis particularly when identified at an advanced metastatic stage [84]. The involvement of SIRT7 in this malignancy seems intricate and likely contingent on the specific context. Elevation in SIRT7 levels within BCa tumors compared to healthy tissues was described [85, 86]. However, a contrasting pattern emerges in high-grade and invasive BCa cases, where SIRT7 levels exhibit a reduction as compared to low grade tumors, suggesting a different influence of SIRT7 in tumor progression at different stages of this malignancy [85, 86]. Intriguingly, the role of SIRT7 in BCa cells proliferation appears to be contingent on the specific cell as it can both stimulate or inhibit cell proliferation depending on the specific cell type [85, 86] (Fig. 7A and Table 1). Divergent findings have emerged concerning the impact of SIRT7 on migration and invasion of BCa cells. In line with the observed decline in invasive cancers, *SIRT7* depletion heightens the migration and invasion of BCa cells by fostering EMT through dampening E-Cadherin (*CHD1*) expression. This is mechanistically driven by increased acetylation and overall levels of the histone methyltransferase EZH2, leading to the EZH2-mediated deposition of repressive histone mark H3K27me3 (trimethylation of histone H3 at lysine 27) at the *CHD1* gene promoter, consequently suppressing its expression (Fig. 7A and Table 1) [85]. In stark contrast, another investigation has paradoxically demonstrated that SIRT7 depletion dampens the migratory capability of BCa cells, although the mechanisms underlying this phenomenon remain uncharacterized (Fig. 7A and Table 1) [86]. To ultimately define the precise role of SIRT7 in bladder cancer, more comprehensive in vivo studies are essential.

### SIRT7 in sarcoma

Sarcomas, tumors presumed to emerge from mesenchymal origins, account for approximately 21% of pediatric and less than 1% of adult solid malignant cancers [87]. They can be broadly categorized into two main groups: bone sarcomas and soft tissue sarcomas. Soft tissue sarcomas generally take shape within muscles, joints, fat, nerves and blood vessels. On the contrary, bone sarcomas primarily encompass osteosarcomas and Ewing’s sarcomas. These malignancies are frequently associated with unfavorable prognoses, largely attributed to their tendency for diagnosis at advanced stages [87].

In fibrosarcoma cells, SIRT7 assumes a critical function in sustaining tumor growth through epigenetic repression of pivotal tumor suppressors including RPs *via* H3K18 deacetylation [22] (Fig. 7B and Table 1). Moreover, SIRT7 stimulates the expression of pro-metastatic factors, such as MMP16 and VEGF-A, consequently fostering cell migration and facilitating the formation of metastases (Fig. 7C and Table 1) [81].

SIRT7 also demonstrates pro-tumorigenic attributes within osteosarcoma cells. Levels of SIRT7 show an elevation within these tumors when compared to healthy tissues and exhibit an inverse correlation with patient survival [39]. The depletion of *SIRT7* in these cells diminishes proliferation, invasion and migration in vitro, and it restricts tumor growth in mouse xenograft models [39]. Mechanistically, SIRT7 represses the expression of the tumor suppressor *CDC4* through deacetylation of H3K18 (Fig. 7D and Table 1) [39].

### Role of SIRT7 in breast cancer

**Fig. 8:**
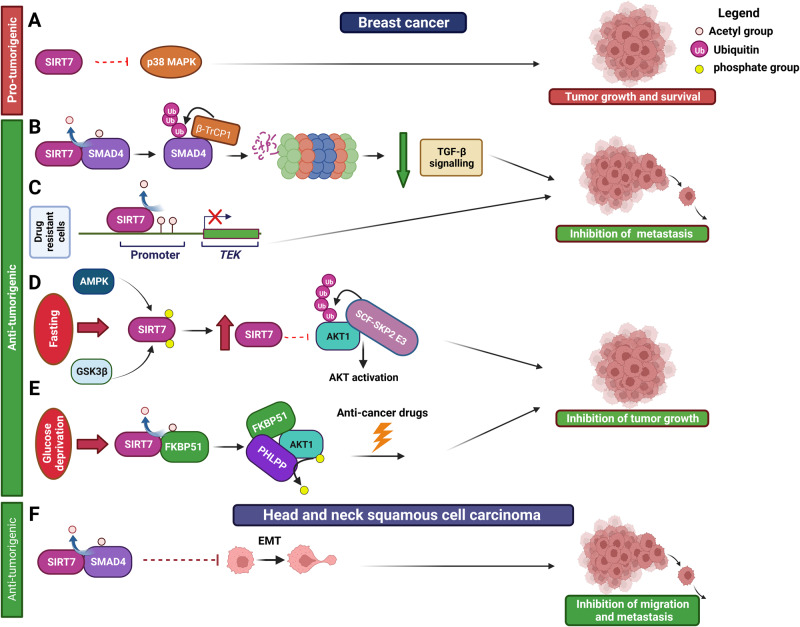
Role of SIRT7 in breast cancer and head and neck squamous cell carcinoma. See text for details.

Breast cancer is a heterogeneous disease and stands as the most prevalent malignancy among women. In its initial stages, the prognosis for breast cancer is favorable. However, the scenario takes a bleak turn when the disease is identified at a metastatic stage [88]. SIRT7 levels increase in different stages of breast cancer but decline in metastasis derived from this malignancy [89–91]. This pattern implies that SIRT7 might wield distinct functions across various stages of breast cancer progression. However, conflicting findings have arisen regarding the influence of SIRT7 in breast cancer. Studies demonstrated that depletion of *SIRT7* within breast cancer cell lines yields significant reductions in both proliferation and migration rates in vitro and in vivo, an effect at least partially attributed to the activation of the p38 MAPK signaling cascade (Fig. 8A and Table 1) [92, 93]. In stark contradiction to these results, another study demonstrated that depletion of SIRT7 promotes breast cancer cells growth in mouse xenografts models [89]. Considering that these studies have utilized consistent cellular models, additional investigation is warranted to comprehensively elucidate the underlying conditions that have contributed to the emergence of these disparate outcomes.

SIRT7 appears to counteract development of metastasis originating from breast cancer as demonstrated by xenografts experiments and using transgenic mouse models of breast cancer [89]. Mechanistically, SIRT7 operates by deacetylating SMAD4. This process triggers the ubiquitination of SMAD4 by the ubiquitin ligase β-TrCP1, leading to the proteasomal degradation of SMAD4. As a result, SIRT7 weakens the transforming growth factor beta (TGF-β) signaling cascade, effectively curbing EMT and the formation of metastases (Fig. 8B and Table 1) [89, 90]. Intriguingly, the effect of SIRT7-mediated deacetylation of SMAD4 appears to exert tumor-suppressive roles in breast cancer and, as illustrated later, in oral squamous tumors [94] which sharply differ from its function in melanoma and prostate cancer [69, 83]. This aligns with dual roles of SMAD4 across malignancies and in different stages of tumor progression [89]. This evidence illustrates how SIRT7 may exert opposite pro-tumorigenic or tumor-suppressive functions across different malignancies by controlling the same signaling pathway.

SIRT7 plays also a role in suppressing the metastasis originating from doxorubicin-resistant breast cancer cells [40]. Cancer cells that have developed resistance to this drug tend to exhibit increased migratory capacities and a higher likelihood of forming metastases. SIRT7 seems to counteract metastasis in these cells by epigenetically repressing the expression of the tyrosine kinase receptor TIE2/TEK *via* H3K18 deacetylation at the gene promoter (Fig. 8C and Table 1) [40]. Additionally, SIRT7 plays and indispensable role in promoting anti-tumor effects of intermittent fasting [71]. Fasting induces a comprehensive shift in metabolites and growth factors, which seems to diminish the resilience of cancer cells, thereby enhancing the effectiveness of anti-cancer therapies [95]. In response to nutrient scarcity, the activation of AMP-activated protein kinase (AMPK), a pivotal player in sensing energy shortage, triggers the initial phosphorylation of SIRT7. This crucial event primes the stage for subsequent phosphorylation by glycogen synthase kinase 3β. This orchestrated sequence effectively halts the ubiquitination process and curtails proteasomal-driven degradation of SIRT7, ultimately leading to a surge in its protein levels. Upon stabilization, SIRT7 assumes the role of restraining the polyubiquitination process of AKT orchestrated by the SCF-SKP2 E3 ligase complex. Given that the polyubiquitination of AKT facilitates its localization to the cell membrane to trigger its activation, through this mechanism SIRT7 effectively curtails the AKT signaling cascade, which, in turn, plays a pivotal role in fueling cancer cell survival, driving the progression of cancer (Fig. 8D and Table 1) [71]. SIRT7 employs an additional mechanism to inhibit AKT signaling in breast cancer cells. More precisely, SIRT7 takes part in deacetylating the scaffolding protein FKBP51, which, in turn, promotes the interaction between the PH domain leucine-rich repeat protein phosphatase (PHLPP) and AKT. This process allows for PHLPP-dependent dephosphorylation and subsequent inactivation of AKT, effectively counteracting AKT-driven pro-tumorigenic functions. Given that glucose deprivation significantly triggers this pathway, it was proposed that treatment with the glucose analog 2-Deoxy-D-glucose (2DG), which mimics glucose deprivation, maybe used as a chemo-sensitizing agent (Fig. 8E and Table 1) [96]. Collectively, this evidence strongly indicates that the SIRT7-AKT axis could serve as a pivotal signaling cascade underpinning the advantageous outcomes of tailored dietary regimens in combination with chemotherapy for breast cancer. This axis presents a compelling avenue for devising novel therapeutic approaches to combat this malignancy. Remarkably, SIRT7 plays an opposite effect in the activation of the AKT signaling cascade in other malignancies such as lung and thyroid cancers [73, 76]. Thus, investigating the potential development of anti-cancer drugs targeting the SIRT7-AKT pathway seems to hold promise, so far, only in this specific malignancy. Interestingly, during glucose deprivation, the phosphorylation of SIRT7 by AMPK leads to the exclusion of SIRT7 from the nucleolus. This event serves to limit pre-RNA synthesis and maturation when nutrient availability is scarce [1]. Consequently, beyond its role in regulating the AKT signaling pathway, intermittent fasting might impede cell proliferation by reducing SIRT7’s influence on ribosome biogenesis. Additionally, as previously mentioned, glucose deprivation appears to enhance SIRT7 mono-ADP ribosylation, activate the SIRT7-macroH2A1 pathway and probably favor mono-ADP ribosylation of still unknown targets [5]. Therefore, SIRT7 may exert anti-tumorigenic functions in breast cancer by controlling these pathways in response to specific dietary regimens.

### SIRT7 in head and neck squamous cell carcinoma (HNSCC)

HNSCCs mainly derive from the mucosal epithelium of the oral cavity, pharynx and larynx [97]. Intriguingly, SIRT7 appears to be a potent tumor suppressor in HNSCC. Consistently. SIRT7 levels are reduced in these tumors as compared to healthy tissues and this event correlates with enhanced formation of lymph node metastasis [98]. Similarly to breast cancer, in these tumors, SIRT7 inhibits migration and metastasis formation by deacetylating and inhibiting SMAD4, thus reducing EMT (Fig. 8F and Table 1) [94].

### SIRT7 in hematologic malignancies

The potential tumor-suppressive role of SIRT7 extends its reach to encompass hematologic malignancies. Recent investigations have shed light on a discernible decline in SIRT7 levels observed in acute myeloid leukemia (AML) and chronic myeloid leukemia (CML) when contrasted with levels in healthy donors. Intriguingly, within the patient cohort, a positive response to therapy correlated with a rise in SIRT7 levels, while patients experiencing progression or relapse displayed a corresponding decrease. Equally noteworthy, the pharmacological restraint of key oncogenic drivers in these malignancies triggered an enhancement in SIRT7 expression [99]. These compelling findings suggest that SIRT7 could potentially wield substantial tumor-suppressive influence, at least within a subset of hematologic malignancies. However, rigorous further exploration is indispensable to validate this hypothesis.

## Conclusions

SIRT7 plays a complex role in both the initiation and progression of tumors. It is evident that SIRT7 acts to counteract tumor formation in response to carcinogens and oncogenes in vivo, aligning with its crucial function in maintaining genomic stability. However, once cells acquire a malignant phenotype, SIRT7 appears to exert either tumor-suppressive or pro-oncogenic functions in a context-dependent manner.

Notably, the dualistic role of SIRT7 is observed not only across a spectrum of malignancies but also within distinct tumor subtypes and at different phases of tumor progression. Genetic alterations, such as specific oncogene activation or tumor suppressor inactivation, can significantly modulate the impact of SIRT7 on tumor progression.

In comparison to other members of the sirtuin family, SIRT7 stands out due to its unique functions. These encompass the control of nucleolar functions, alteration of ribosomal composition with potential impacts on mRNA translation, regulation of mRNA processing and transcription, and distinctive catalytic activities toward specific targets implicated in tumor progression. Notably, SIRT7’s ability for auto-monoADP ribosylation is intriguing; this autocatalytic activity significantly influences its chromatin distribution and is strongly influenced by glucose availability. Consequently, SIRT7 may play a crucial role in influencing epigenetic regulation of gene expression, especially in conditions of nutrient scarcity often observed in cancer cells.

Additionally, SIRT7 exhibits a unique feature of interacting with specific targets in a RNA-dependent manner, and the binding of RNA stimulates its catalytic activity. In specific cancer cells, the abundance of specific RNA may prominently influence SIRT7 interactions and activities, ultimately influencing tumorigenesis.

The emerging role of SIRT7 as a multifaceted player in cancer highlights its significance as a potential diagnostic marker, prognostic indicator, and therapeutic target. As our understanding of SIRT7 functions continues to evolve, unraveling its molecular intricacies within the context of various cancers holds promise for advancing personalized cancer treatments tailored to specific malignancies.
